# Whole genome sequencing for investigations of meningococcal outbreaks in the United States: a retrospective analysis

**DOI:** 10.1038/s41598-018-33622-5

**Published:** 2018-10-25

**Authors:** Melissa J. Whaley, Sandeep J. Joseph, Adam C. Retchless, Cecilia B. Kretz, Amy Blain, Fang Hu, How-Yi Chang, Sarah A. Mbaeyi, Jessica R. MacNeil, Timothy D. Read, Xin Wang

**Affiliations:** 1 Meningitis and Vaccine Preventable Diseases Branch, Centers for Disease Control and Prevention, Atlanta, Georgia USA; 2 Division of Infectious Diseases, Department of Medicine, Emory University School of Medicine, Atlanta, Georgia USA

## Abstract

Although rare in the U.S., outbreaks due to *Neisseria meningitidis* do occur. Rapid, early outbreak detection is important for timely public health response. In this study, we characterized U.S. meningococcal isolates (N = 201) from 15 epidemiologically defined outbreaks (2009–2015) along with temporally and geographically matched sporadic isolates using multilocus sequence typing, pulsed-field gel electrophoresis (PFGE), and six whole genome sequencing (WGS) based methods. Recombination-corrected maximum likelihood (ML) and Bayesian phylogenies were reconstructed to identify genetically related outbreak isolates. All WGS analysis methods showed high degree of agreement and distinguished isolates with similar or indistinguishable PFGE patterns, or the same strain genotype. Ten outbreaks were caused by a single strain; 5 were due to multiple strains. Five sporadic isolates were phylogenetically related to 2 outbreaks. Analysis of 9 outbreaks using timed phylogenies identified the possible origin and estimated the approximate time that the most recent common ancestor emerged for outbreaks analyzed. U.S. meningococcal outbreaks were caused by single- or multiple-strain introduction, with organizational outbreaks mainly caused by a clonal strain and community outbreaks by divergent strains. WGS can infer linkage of meningococcal cases when epidemiological links are uncertain. Accurate identification of outbreak-associated cases requires both WGS typing and epidemiological data.

## Introduction

Meningococcal disease is a serious and life-threatening bacterial infection, caused by *Neisseria meningitidis* (Nm), with a case fatality ratio of 10–15%; 11–19% of survivors experience long-term sequelae^1^. Most meningococcal disease cases are sporadic in the United States. However, a number of outbreaks occurred in different settings, such as the recent meningococcal serogroup B (NmB) outbreaks on university campuses and meningococcal serogroup C (NmC) outbreaks among the general population and among men who have sex with men (MSM)^2–8^. Large meningococcal outbreaks and epidemics have also been reported in other countries^9–15^.

Rapid and early detection of outbreaks is critical to guide prompt and appropriate public health interventions to control the outbreaks and prevent additional cases. Molecular typing has played an important role in outbreak investigations, identifying outbreak strains by assessing their genetic relatedness and providing evidence to understand case transmission and linkage. Historically, typing with pulse field gel electrophoresis (PFGE) has been the gold standard for many infectious diseases, including meningococcal disease^2,16^. Many meningococcal reference laboratories also utilize multilocus sequence typing (MLST)^17^ and genotyping of outer membrane proteins (PorA^18^ and FetA^19^) to determine the lineage and genotype of the outbreak strains^2,20^. However, disadvantages have been described for both methods. PFGE is time consuming and labor intensive. Standardization between laboratories is challenging, leading to irreproducibility of PFGE data^21^. The interpretation of strain relatedness based on PFGE patterns can be subjective and discordant because it lacks the discriminatory capacity and phylogenetic basis of more advanced methods^22^. Isolates with high genetic similarity may appear different in PFGE analysis^23,24^. While Sanger sequencing-based typing using MLST genes and genes coding for outer membrane proteins is useful to define bacterial molecular epidemiology and population structure, it does not provide sufficient discriminatory power to differentiate outbreak strains from sporadic strains or strains with high genetic similarity. Analyses of MLST data often assume that isolates sharing the same sequence type constitute an evolutionary unit, but this assumption is over simplified for species such as Nm, which has higher rates of recombination^25,26^.

With rapid advances in next-generation sequencing (NGS) during recent years, the cost of whole genome sequencing (WGS) has dropped dramatically. The rapid turnaround time and availability of automated WGS analysis tools make it possible to use this technology for outbreak investigation and surveillance of infectious disease^27–29^. WGS can distinguish strains based on differences in single nucleotide polymorphisms (SNPs) or multilocus genes across the whole genome, an essential feature needed in Nm outbreak investigations when strains show high genetic similarity. WGS has been successfully used to elucidate the evolution of some Nm subtypes^11,30–35^, to assess the epidemiological association between Nm carriage and disease-causing isolates^36^ as well as for outbreak investigations for Nm^32^ and other pathogens such as *N. gonorrhoeae*^37^.

Before implementing WGS in routine surveillance and outbreak investigations for Nm, it is essential to evaluate WGS-based methods in comparison with traditional typing methods to establish the suitable scheme for the outbreak investigation of meningococcal diseases. This study aimed to re-characterize NmB and NmC outbreaks in the United States during 2009–2015 using WGS-based methods and to develop an optimal WGS-based typing scheme for Nm outbreak investigations.

## Results

### Molecular features of Nm outbreak isolates

A total of 19 unique strain genotypes, serogroup:MLST sequence type (ST)/clonal complex (CC):PorA type:FetA type, were detected among isolates from 15 outbreaks: 10 genotypes among 8 NmB and 9 among 7 NmC outbreaks (Table 1). While most outbreaks had a unique genotype within an outbreak, multiple genotypes were detected in 5 outbreaks (NmB: OB3, OB6, and OB10; NmC: OB7 and OB9). The strain genotypes B:ST32/CC32:P1.7,16–20:F3–3 and C:ST-11/CC11:P1.5–1,10–8:F3–6 were the most common, each causing more than 1 outbreak (NmB: OB6, OB11, and OB12; NmC: OB4, OB7, OB8, and OB9). Deletions and frameshifts were observed in *porA* and *fetA* among outbreak isolates; these changes have been previously reported to occur in NmB and NmC isolates^38^

**Table 1:** Characteristics of 2009–2015 outbreak isolates.

Outbreak	Genotype (Serogroup:MLST ST/CC:porA type:fetA type) [number of isolates per genotype]	Outbreak PFGE pattern type	Outbreak year	Outbreak population
OB1	B:ST-283/CC269:P1.19-1,15-11:F5-9 [4]	317 and 318	2009	Organization
OB2	C:ST-32/CC32:P1.7,16:F3-3 [5]	483 and 484	2009–2010	Community
OB3	B:ST-269/CC269:P1.22,2-9:F5-1 [2]	347	2010	Organization
	B:ST-269/CC269:P1.22,9:F5-1 [1]	347		
OB4	C:ST-11/CC11:P1.5-1,10-8:F3-6^a^ [4]	37	2010	Organization
OB5	C:ST-11/CC11:P1.22-1,14:F3-6 [8]	91	2010	Organization
OB6	B:ST-9313/CC32:P1.7,16-20:F3-3 [1]	187	2011	Community
	B:ST-32/CC32:P1.7,16-20:F3-3^b^ [1]	187		
	B:ST-9314/CC41/44:P1.7-1,1:F3-29 [1]	312		
OB7	C:ST-11/CC11:P1.5-1,10-8:F3-6^a^ [11]	265, 383, 417, 428, 430, 459, and 466	2011–2013	Community
	C:ST-11/CC11:P1.5-1,10-8:DI^c^ [1]	454		
	C:ST-11/CC11:P1.5-1,10-35:F3-6 [1]	439		
	C:ST-11/CC11:P1.5-1, 10-1:DI [1]	437		
OB8	C:ST-11/CC11:P1.5-1,10-8:F3-6^a^ [5]	265, 366, and 414	2012–2013	Community
OB9	C:ST-11/CC11:P1.5-1,10-8:F3-6^a^ [2]	265 and 413	2012–2013	Community
	C: ST-11/CC11:P1.5,2:F3-3 [5]	415		
	C:ST-11/CC11:DI: F3-3 [2]	415		
OB10	B:ST-409/CC41/44:P1.5-1,2-2:F1-5 [7]	429	2013	Organization
	B:ST-10563/CC41/44:P1.17,9:F1-5 [1]	441		
OB11	B:ST-32/CC32:P1.7,16-20:F3-3^b^ [4]	467 and 468	2013	Organization
OB12	B:ST-32/CC32:P1.7,16-20:F3-3^b^ [6]	467	2015	Organization
OB13	B:ST-9069/unassigned CC:P1.7-2,4:F1-5 [2]	516 and 517	2015	Organization
OB14	C:ST-11/CC11:P1.18-1,3:F3-6 [7]	428	2015	Community
OB15	B:ST1624/CC167:P1.5-1,10-1:F3-4 [2]	393	2011	Organization

^a^Genotype observed in multiple NmC outbreaks (OB4, OB7, OB8, and OB9).

^b^Genotype observed in multiple NmB outbreaks (OB6, OB11, and OB12).

^c^DI indicates deletion within gene.

.

Among the outbreak isolates, 30 unique PFGE patterns, 12 for NmB and 18 for NmC, were identified (Table 1, Supplementary Figs 1 and 2). One to two PFGE patterns per NmB outbreak were identified, while 1–10 patterns were identified per NmC outbreak. Although 90% of PFGE patterns were unique to a specific outbreak, some PFGE patterns were seen in multiple outbreaks.

### WGS typing methods for phylogenetic classification of Nm outbreak isolates

**Figure 1:**
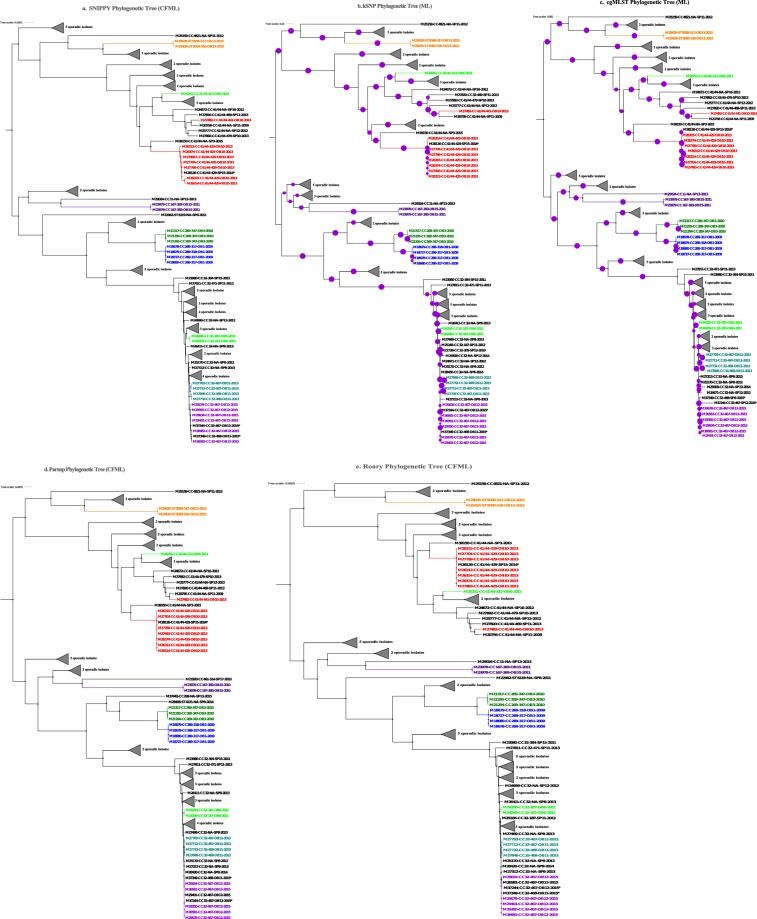
Phylogenetic trees generated using different WGS analysis methods for NmB outbreak and sporadic isolates, 2009–2015. (**a**) SNIPPY Phylogenetic Tree reconstructed from a whole genome core alignment generated based on reference-based short read mapping and corrected for recombination using ClonalFrameML. (**b**) kSNP Phylogenetic Tree, a maximum likelihood (ML) phylogenetic tree generated using RaXML from the core SNP alignment generated by kSNP. (**c**) cgMLST Phylogenetic Tree, an ML tree created from the concatenated core gene alignment using RaXML. (**d**) Parsnp Phylogenetic Tree reconstructed from a whole genome core alignment generated using Parsnp and corrected for recombination using ClonalFrameML. (**e**) Roary Phylogenetic Tree generated from the concatenated core gene alignment; recombination was corrected using ClonalFrameML. Purple circles represent branch-level bootstrap support out of 100 bootstrap estimates. The circumference of a circle is proportional to the bootstrap support.  represented 100% bootstrap support. Bootstrap support was estimated only for kSNP and cgMLST ML phylogenetic trees; the Bayesian recombination-adjusted phylogenies from ClonalFrameML do not estimate bootstrap values. Isolate label contains isolate ID, CC, PFGE pattern, outbreak or sporadic isolates, and year. Geographically matched outbreak and sporadic isolates are indicated by same number following “SP” or “OB” in label. Each colored branch and isolate label represents a different outbreak.

**Figure 2:**
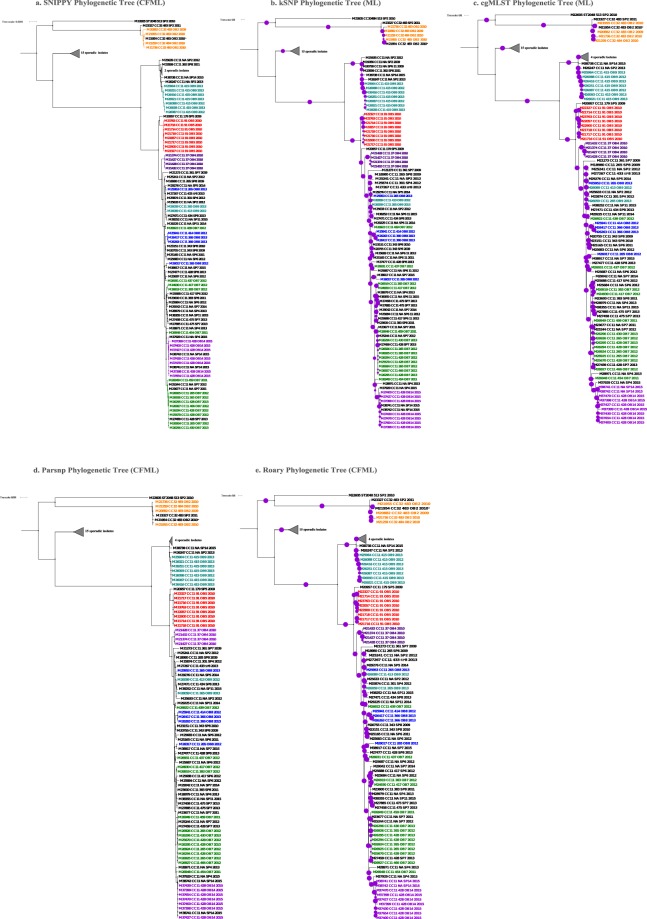
Phylogenetic trees generated using different WGS analysis methods for NmC outbreak and sporadic isolates, 2009–2015. (**a**) SNIPPY Phylogenetic Tree reconstructed from a whole genome core alignment generated based on reference-based short read mapping and corrected for recombination using ClonalFrameML. (**b**) kSNP Phylogenetic Tree, an ML phylogenetic tree generated using RaXML from the core SNP alignment generated by kSNP. (**c**) cgMLST Phylogenetic Tree, an ML tree created from the concatenated core gene alignment. (**d**) Parsnp Phylogenetic Tree reconstructed from a whole genome core alignment generated using Parsnp and corrected for recombination using ClonalFrameML. (**e**) Roary Phylogenetic Tree generated from the concatenated core gene alignment; recombination was corrected using ClonalFrameML. Purple circles represent branch-level bootstrap support out of 100 bootstrap estimates. The circumference of a circle is proportional to the bootstrap support.  represented 100% bootstrap support. Bootstrap support was estimated only for kSNP and cgMLST ML phylogenetic trees; the Bayesian recombination-adjusted phylogenies from ClonalFrameML do not estimate bootstrap values. Isolate label contains isolate ID, CC, PFGE pattern, outbreak or sporadic isolates, and year. Geographically matched sporadic and outbreak isolates are indicated by the same number following “SP” or “OB” in label. M26263 and M26417 were isolates from the same person. Each colored branch and isolate label represents a different outbreak.

The 6 WGS analysis methods (Table 2) demonstrated high discriminatory power for classifying NmB and NmC outbreak isolates (Figs 1 and 2) phylogenetically or by genetic distance measures (Supplementary Tables 1 and 2). Isolates from each of the 6 NmB outbreaks (OB1, OB3, OB11, OB12, OB13, and OB15) and 4 NmC outbreaks (OB2, OB4, OB5, and OB14) were grouped into distinct phylogenetic outbreak clades. Isolates from each of the remaining 2 NmB (OB6 and OB10) and 3 NmC outbreaks (OB7, OB8, and OB9) were scattered into more than one phylogenetic clade. Direct qualitative comparison showed that topologies of the phylogenetic trees reconstructed by various WGS analysis methods were similar to each other. Quantitative pairwise tree distance measures using weighted Robinson-Foulds (wRF) showed high similarity between methods that accounted for recombination (SNIPPY, Roary and Parsnp; mean wRF distances were 0.292 and 0.002 for NmB and NmC respectively) and between methods that did not account for recombination (kSNP and cgMLST; mean wRF distances were 0.619 and 0.362 for NmB and NmC respectively). The average pairwise wRF distances between the trees reconstructed with and without accounting for recombination for NmB and NmC were 4.503 and 2.069 respectively (Supplementary Table 3).

**Table 2:** WGS analysis methods evaluated in this study.

WGS analysis method	Type of input data	Reference-based method	Adjusted for recombination or not (method used)	Whole genome alignment length before recombination correction (bps)	Number of nucleotide positions available for phylogenetic reconstruction after recombination correction (bps)
kSNP (k-mer–based)	Contigs	Reference free	No		
Roary	Gene annotations (from contigs)	Reference free	Yes (ClonalFrameML)	NmB – 1117836; NmC–1347579	NmB – 237583; NmC–496020
Parsnp	Contigs	Reference-based	Yes (ClonalFrameML)	NmB – 1331883; NmC–1547169	NmB −265861; NmC–503939
SNIPPY.(hq SNP-based)	Raw reads	Reference-based	Yes (ClonalFrameML)	NmB – 2283986; NmC–2200611	NmB – 605744; NmC–827012
cgMLST	Contigs	Reference-based (core genome MLST genes)	No		
MASH (k-mer–based)	Raw reads	Reference free	No		
BEAST	Parsnp/SNIPPY alignment	Reference-based	Yes (ClonalFrameML)	NmB (CC32 isolates; n = 24) – 2127589; NmC (CC11 isolates; n = 71) – 1764302	NmB – 1709526; NmC –1168741

The intra-outbreak genetic distances ranged from 0 to 186 SNPs/allele differences (Supplementary Table 1). The maximum intra-outbreak SNP differences were less than or equal to the minimum inter-outbreak SNP differences for the recombination-adjusted methods (SNIPPY, Parsnp and Roary). Although the range of estimated intra-and inter-outbreak SNP/allele differences for methods without recombination adjusted (kSNP and cgMLST) were higher than the recombination-adjusted methods, they are generally proportional (Supplementary Table 1). For kSNP and cgMLST, we also observed overlap between the maximum intra outbreak SNP/allele differences and the minimum inter outbreak SNP/allele differences for a few outbreaks (Supplementary Table 1).

The pairwise genetic distance measures (SNP/allele differences) between outbreak and sporadic isolates were estimated. The mean genetic distances (SNP/allele differences) between outbreak isolates from a single phylogenetic clade and any sporadic isolates ranged between 209 and 1418 SNPs/alleles, depending on the method (Supplementary Fig. 3). The maximum dissimilarity measures within NmB and NmC outbreaks by MASH were 0.04% and 0.09%, respectively, while the average dissimilarity measure observed between outbreak isolates and any sporadic isolates were 1.83%.

### Comparison of PFGE and WGS for assessing genetic relatedness of Nm outbreak isolates

Based on wRF phylogenetic tree distances, we observed two distinct groups of WGS typing methods, recombination-adjusted methods (SNIPPY, Parsnp, and Roary) and methods that do not account for recombination (kSNP and cgMLST). We focused on one recombination-adjusted method (SNIPPY) and a method that does not account for recombination (cgMLST), and compared them with PFGE. cgMLST is widely used for typing meningococcal isolates, and uses *de novo* assembly data. SNIPPY is a reference-based short read mapping method that generates a whole genome alignment with more nucleotide positions, leading to a more efficient recombination correction, compared to the core genome alignment generated by Parsnp and Roary. Of the 8 NmB outbreaks analyzed, the results of PFGE and WGS were in good agreement on 2 (OB15 and OB3) but gave discordant results on the other 6 outbreaks (Fig. 1). As shown in Fig. 1, Supplementary Table 1 and Supplemental Fig. 1, 3 NmB outbreaks had 2 PFGE patterns each (OB13 with PFGE patterns 516 and 517; OB11 with 467 and 468; and OB1 with 317 and 318). However, SNIPPY and cgMLST classified the isolates of each outbreak into a single distinct phylogenetic clusters with a few SNP/allele differences (<17 SNP/allele differences between OB13 isolates, 13–86 between OB11 isolates, and 0–5 between OB1 isolates). Although NmB OB12 isolates had PFGE pattern 467, which is one of the two patterns detected among OB11 isolates, OB11 and OB12 isolates formed phylogenetically distinct clades using SNIPPY and cgMLST. Two of 3 NmB OB6 isolates that had PFGE pattern 187 clustered together, with 1 SNP and 30 allele differences by SNIPPY and cgMLST, respectively. However, the third isolate (M24251) that had PFGE pattern 312 was genetically different from the other two isolates by both PFGE and WGS-based methods, with 706 SNPs and 1486 alleles differences by SNIPPY and cgMLST, respectively. For OB10, 7 of the 8 isolates with PFGE pattern 429 formed a single phylogenetic clade with 12 SNP and 83 allele differences by SNIPPY and cgMLST analysis, respectively. An isolate (M28126) from a case with known epidemiological links to OB10 also had the same PFGE pattern and clustered with the 7 isolates. The remaining OB10 isolate (M27482) had a different PFGE pattern (441) and was genetically different with 475 SNP and 1139 allele differences.

The results of PFGE and WGS were in good agreement on 3 NmC outbreaks (OB4, OB5, and OB14) but discordant on 4 (Fig. 2 and Supplementary Fig. 2). Outbreaks OB2, OB7, OB8, and OB9 were represented by multiple PFGE patterns. OB2 was represented by 2 PFGE patterns (483 and 484), but all OB2 isolates formed a distinct phylogenetic clade by WGS methods. OB7 had the highest variability by PFGE, with 10 patterns detected among 14 isolates. Six OB7 isolates with 3 PFGE patterns (265, 428, and 459) were indistinguishable (100% BioNumerics calculated similarity) and formed a phylogenetic cluster by SNIPPY and cgMLST, while the remaining 8 OB7 isolates (including 1 isolate with pattern 428) were intermixed with OB8 isolates and were scattered randomly on the phylogenetic tree. SNP/allele differences among OB7 ranged from 0 to 201 (Fig. 2; Supplementary Table 1). All 5 OB8 isolates were represented by 3 PFGE patterns and showed 0–267 SNP/allele differences by SNIPPY and cgMLST. WGS methods grouped 3 OB8 isolates into a tight phylogenetic group with 0–50 SNP/allele differences (Supplementary Table 1), while the remaining 2 were scattered on the phylogenetic tree (Fig. 2). Seven out of 9 OB9 isolates had the PFGE pattern 415, and WGS methods consistently clustered those 7 isolates into a phylogenetic group; the remaining 2 OB9 isolates with different PFGE patterns (413 and 265) were genetically distinct from the other 7 OB9 isolates and clustered with sporadic isolates.

### Tracing the origin of outbreak isolates using timed phylogenetic analysis

**Figure 3:**
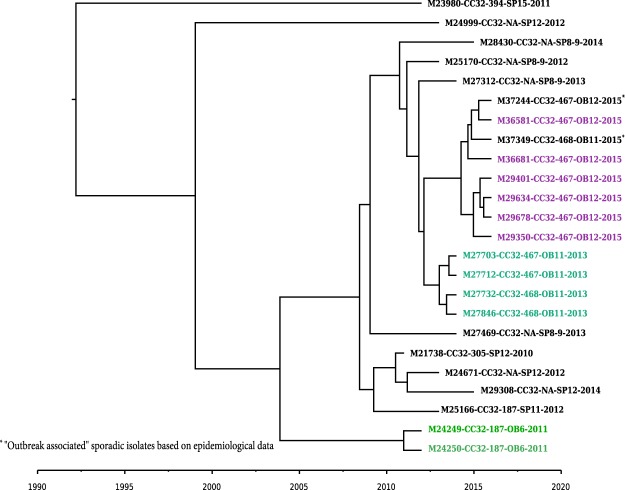
Timed phylogenetic tree of NmB CC32 outbreak and sporadic isolates. A time-dependent phylogenetic reconstruction of the NmB CC32 outbreak and sporadic isolates, inferred by Bayesian inference using BEAST. The NmB CC32 consisted of 24 isolates from 3 outbreaks (OB6, OB11, and OB12), along with the geographically matched sporadic isolates. Geographically matched sporadic and outbreak isolates have the same number following “SP” or “OB” in label. Isolate label contains isolate ID, CC, PFGE pattern, outbreak or sporadic isolates, and year. Each colored branch and isolate label represents a different outbreak.

In order to assess the evolutionary relationship between strains causing different outbreaks, and to determine potential linkage between outbreaks, we performed BEAST analysis on a subset of NmB and NmC isolates and reconstructed a timed phylogeny to estimate the time to the most recent common ancestor from which each isolate per outbreak was derived. From the BEAST analysis, we estimated the substitution rate for NmB and NmC isolates as 4.6 × 10^−6^ (95% highest posterior density (HPD) ranging from 2.2 × 10^−6^–7.8 × 10^−6^) and 4.9 × 10^−6^ (95% HPD ranging from 3.1 × 10^−6^–6.9 × 10^−6^) substitutions/site/year respectively, which was similar to previous estimates in *N. meningitidis*^35^ and the related species *N. gonorrhoeae*^37^. The timed phylogeny for the 24 NmB CC32 isolates from outbreaks (OB11, OB12, and OB6) and sporadic cases is shown in Fig. 3. All OB11 isolates from 2013 were derived from a common ancestor that may have appeared in early 2012. Isolate M37349 was obtained from a case reported in 2015 from the same state where the OB11 outbreak occurred, and was originally considered an OB11-associated isolate because of the epidemiological link. Based on the molecular clock analysis, the ancestor of this isolate was estimated to have emerged in early to mid-2013, differently from the common ancestor of the OB11 isolates that emerged in 2012. Moreover, this OB11-associated isolate (M37349) was phylogenetically more similar to OB12 isolates, with a maximum of 10 SNP differences from the OB12 isolates, compared to a 21 SNP difference from the OB11 isolates based on recombination-corrected SNIPPY analysis (Supplementary Table 1). Isolate M37244, collected from an OB12-associated case, might have been part of OB12, as the emergence of its common ancestor is the same as that of OB12 isolates. BEAST analysis also confirmed that only 2 (M24249 and M24250) of the 3 OB6 isolates were derived from a common ancestor (Fig. 3 and Supplementary Table 1).

**Figure 4:**
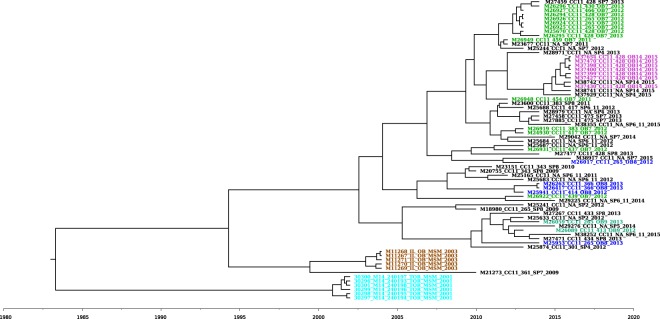
Timed phylogenetic tree of NmC CC11 outbreak and sporadic isolates. A time-dependent phylogenetic reconstruction of the NmC CC11 outbreak and sporadic isolates, inferred by Bayesian inference using BEAST. The NmB CC11 consisted of 71 isolates from 6 outbreaks (OB7, OB8, OB9, OB14, Chicago (2003), and Toronto (2001)), along with the geographically matched sporadic isolates (SP7, SP8, SP9, and SP14). M26263 and M26417 were isolates from the same person. Each colored branch and isolate label represents a different outbreak.

The timed phylogenetic tree reconstructed using BEAST for CC11 NmC isolates in the US MSM population, along with phylogenetic related sporadic and outbreak isolates, is shown in Fig. 4. The 2 NmC CC11 outbreaks, reported in 2001 in Toronto and 2003 in Chicago, clustered into 2 distinct phylogenetic clades; isolates within each clade shared a common ancestor. Based on the timed phylogeny, 10 out of the 14 OB7 isolates and all the OB14 isolates were derived from the same common ancestor, indicating that the 2 outbreaks might be related. The remaining 4 OB7 isolates were phylogenetically mixed with sporadic isolates from the same and different geographical locations. Interestingly, 3 geographically matched sporadic isolates (M27459, M25244, and M23677) were found to be derived from the same ancestral node as 9 of the 10 phylogenetically related OB7 isolates. When these 3 sporadic isolates were analyzed together with the 9 OB7 isolates, there were 12 SNP/57 allele differences found by SNIPPY and cgMLST, respectively, indicating that the 3 sporadic isolates were genetically very similar to OB7 isolates (Supplementary Table 1). All 7 OB14 isolates were phylogenetically grouped together, along with 2 additional geographically matched sporadic isolates (M38742 and M38741) using all 6 WGS analysis methods. The BEAST analysis indicated that the OB14 isolates and the phylogenetically related sporadic isolates were derived from the same common ancestor, implying the possibility that OB14 isolates might have been evolved from these 2 sporadic isolates (Fig. 4). In addition, 1 OB7 isolate (M26948) was phylogenetically closer to the OB14 clade than the OB7 clade. Consistent with the phylogenetic analysis described in the PFGE and WGS comparison, 3 out of 4 OB8 isolates (M25941, M26263, and M26417) formed a clade that shared a common ancestor. M26263 and M26417 were isolates from the same patient.

## Discussion

While the incidence of meningococcal disease is at a historic low in the US, outbreaks occurring in communities and organizations remain a public concern. To better understand the genetics of US meningococcal outbreak strains, we characterized the isolates from 15 US outbreaks during 2009–2015 using different molecular typing methods. The majority of the meningococcal outbreak isolates belonged to hyper-invasive lineages CC32, CC41/44, CC269, and CC11^38–40^. With the addition of FetA and PorA types to the 7 gene MLST scheme, US outbreak strain genotypes matched isolates associated with outbreaks reported in Italy and Brazil^39,41,42^. Some were seen among a small proportion, 0.6–8.5%, of US sporadic isolates collected through Active Bacterial Core Surveillance (2000–2014).

Information on the genetic relatedness of meningococcal outbreak strains is used to determine whether an outbreak was caused by clonal or divergent strains, and to predict potential linkage of suspected outbreak-related case when an epidemiological link is not certain. WGS demonstrated high resolution and discriminatory power to assess genetic similarity compared to PFGE and other typing methods. Isolates of a single phylogenetic cluster had multiple PFGE patterns, and phylogenetically distinct isolates had the same PFGE pattern, suggesting PFGE is unable to conclusively establish the genetic relatedness of meningococcal isolates and has limited reliability to assist with epidemiological investigation of meningococcal outbreaks. WGS-based phylogenetic analysis revealed that most of the US meningococcal outbreaks, either organization- or community-based, were caused by clonal strains with a low rate of genetic variation. However, two meningococcal outbreaks (OB7 and OB8) among MSM were each caused by divergent strains. The isolates from each of the 2 MSM outbreaks showed a high degree of intra-outbreak genetic variation compared to non-MSM outbreaks.

Including geographically matched sporadic isolates in the analysis allows for the understanding of the genetic diversity of meningococcal outbreak strains in relation to non-outbreak strains. Most of the sporadic isolates were not phylogenetically related to the outbreak isolates; however, 3 and 2 sporadic isolates were observed to be tightly clustered with isolates from OB7 and OB14, respectively. These sporadic isolates were collected either during the outbreak or a month before the outbreaks were reported, suggesting some outbreak strains or their common ancestors, once emerged, may be very stable and stay in circulation for a long period of time. In addition, sporadic isolates that are phylogenetically similar to outbreak isolates should be further investigated in order to determine whether they are associated with an outbreak. Using timed-phylogenetic analysis, we were able to trace the potential origin of meningococcal outbreak strains, determine the potential linkage of 2 outbreaks, and understand the possible transmission path of the meningococcal strains. For an example, recent NmC outbreaks among MSM (OB7, OB8, and OB14) did not result from the direct transmission of the 2003 MSM outbreak isolates, although they might share the same common ancestor that emerged around 1994. The 2015 NmB OB12 was either caused by a strain derived from the strain causing the 2013 NmB OB11 in a neighboring state or a strain that diverged from their common ancestor that emerged in 2008.

The performance of each WGS-based typing method was evaluated for ability to clearly distinguish outbreaks from sporadic cases. WGS analysis methods resulted in very comparable phylogenetic topologies and were highly sensitive in distinguishing multiple distinct Nm outbreak clusters. To apply these methods effectively for disease surveillance and outbreak investigation, each method was evaluated for processing time, analysis capacity, and ability to assess genetic diversity introduced through homologous recombination. As homologous recombination is the major driving force for genetic variation and sequence diversity in meningococcal strains, assessment of genetic similarity and transmission of meningococcal strains must consider homologous recombination. Although the topologies of phylogenetic trees by different WGS analysis methods are similar for this dataset, with or without correction for homologous recombination, the differences in wRF tree distance measures could be due to the inflated branch lengths introduced by recombination. A good understanding of strain transmission requires a method that is able to detect genetic recombination and accounts for its evolutionary effects and thereby accurately reconstruct the clonal relationship between meningococcal isolates during an outbreak. Both k-mer–based methods, MASH and kSNP, seem feasible; they are extremely fast and require fewer computational resources. However, k-mer–based methods have inherent drawbacks^43^. A recent study^43^ indicated that k-mer–based methods especially MASH might suffer from relying on missing k-mers; due to erroneous *de novo* assembly artifacts, the presence of mobile elements or contamination is treated as informative and, thus, will result in biased distance estimates. In addition, model-based recombination detection is not feasible for kSNP core SNP alignment, which may overestimate the number of SNP differences between isolates. Furthermore, unlike MASH, kSNP is not scalable for analyzing thousands of genomes simultaneously^43^.

Methods based on *de novo* assembly (Parsnp) and those using gene annotations inferred from *de novo* assemblies (cgMLST and Roary) also showed high accuracy. However, methods that rely on *de novo* assemblies to generate the whole genome core alignment (Parsnp, cgMLST, and Roary) may have lower resolution for SNP detection, because mutations are less likely to emerge in core genes compared to the accessory genes and intergenic regions; these methods therefore may not provide the same resolution as reference-based mapping^44,45^. While Parsnp generated a whole genome alignment quickly, its scalability to analyze thousands of genomes at once is limited^43^. cgMLST treats all genetic variation within a single locus count as a single allelic difference and lacks the implementation of model-based recombination detection. The genetic similarity between any 2 isolates can be based on the allele differences obtained from cgMLST or the SNP differences obtained from SNP-based analysis; however, the quantified differences detected by these methods are relative and cannot be compared. A recent evaluation of phylogenetic reconstruction methods using bacterial genomes suggested cgMLST-based methods had limited resolution and inference of intra-cluster distances^46^. In general, SNP-based typing provides higher resolution than annotation-based methods and is faster to implement. However, the cgMLST scheme allows standardization of sequencing data. Roary, unlike cgMLST, which requires a curated set of reference core genes, identified the core genes present among all the Nm isolates analyzed and generated a core gene alignment. SNIPPY, the reference-based short read mapping method, is fast and accurate, and identifies high-quality SNPs. Short-read mapping provides greater sensitivity and specificity than SNPs called from assembled *de novo* contigs because the variant call is based on the full information available in the reads, rather than on the assembler and consensus base caller^47^. Systematic evaluation of various phylogenetic reconstruction methods among bacterial genomes showed that the most accurate phylogenetic reconstruction was obtained from an alignment generated by mapping the sequencing reads to a closely related reference genome^46^. It is also shown that phylogeny inferred from an alignment generated by short read mapping to a less closely related *de novo* assembled genome within the group as a reference was similar to the phylogeny using a closely related completed genome^44^. SNIPPY also generates a whole genome alignment that includes both the SNPs identified as well as the invariant sites, which enabled us to perform model-based recombination detection analysis. This was very important to account for accurate phylogenetic analysis of Nm isolates, due to the inherent high recombination rate.

Even though some studies have attempted to establish a threshold based on allele/SNP differences to define an outbreak strain or clone^22,48^, it is rather complex to determine a meningococcal outbreak strain or clone based solely on differences among the number of specific alleles/SNPs. In most cases, isolates from a single outbreak form a tight phylogenetic cluster, with only a few allele/SNP differences within an outbreak; the minimum inter-outbreak allele/SNP difference is much higher than the maximum intra-outbreak difference. However, several outbreaks showed lower minimum inter-outbreak differences than the maximum intra-outbreak difference, increasing the difficulty in establishing an allele/SNP threshold for outbreak strain or clone. In addition, numbers of allele/SNP differences may vary depending on analysis methods, the reference genome selected, SNP calling parameters, and sequencing metrics^44^. Threshold established for strain definition warrants reevaluation over time when any of these parameters change. Outbreak time span is also a factor, as genetic variations accumulate in Nm isolates over time. Therefore, defining an outbreak strain or clone should integrate both allele/SNP differences and phylogenetic analysis. In addition, the timed phylogenies using evolutionary “molecular clock” models will be useful in situations where there is high similarity (i.e., less evolutionary distance) among isolates in different outbreak clusters; translation of the branch lengths to time points informs the estimated time when the most common recent ancestor emerged and sheds light on potential transmission paths^49^.

For rapid analysis of meningococcal strains, we propose a simple comparison method (e.g. a pairwise distance measure derived from kSNP or MASH) as a first step to provide a quick assessment of genetic relatedness between the newly identified isolates and a comprehensive reference collection of meningococcal isolates. An in-depth phylogenetic analysis using an alignment generated based on short read-mapping to the closest reference meningococcal genome will be conducted to compare the new isolates with the ones that are identified as most closely related in the first step, and accounting for homologous recombination. While it is difficult to establish an allele/SNP difference threshold to define an outbreak strain or clone, the differences obtained from the defined outbreaks in this study can be used as reference values for classifying outbreak strains. According to the recently published “Guidance for the Evaluation and Public Health Management of Suspected Outbreaks of Meningococcal Disease”^50^, WGS data are not required to define an outbreak because not all cases will have an available isolate for WGS. However, WGS may be used to exclude cases caused by strains that are genetically different from the predominant outbreak strain^50^.

## Methods

### Strain collection

Meningococcal disease surveillance is conducted through the Nationally Notifiable Diseases Surveillance System (NNDSS). As part of routine surveillance, state health departments are requested to send isolates from meningococcal disease cases in addition to epidemiological data to CDC for further analyses.

In 2014, CDC requested that state and local health departments review all meningococcal disease cases reported to NNDSS from January 1, 2009 to December 31, 2013, to identify cases that occurred as part of an “outbreak”. Outbreaks are defined as 2 or more primary cases of the same serogroup in an organization (defined as affiliation other than shared geographically delineated community, such as school, university, team, nursing home, etc.) in less than three months, or an increase in disease rates in a community^1^. Following this initial call for cases, four additional outbreaks from January 1, 2014 to December 31, 2015, were reported to CDC. Here, we analyzed 15 outbreaks where ≥50% of cases had viable isolates, including 8 serogroup B outbreaks (NmB = 32 isolates) and 7 serogroup C outbreaks (NmC = 52 isolates) from 10 states. A subset of randomly selected isolates (61 NmB and 56 NmC isolates) from “sporadic” cases collected during 2009–2015 in the same states where the 15 outbreaks occurred were included for the comparison between WGS-based methods; the same number following “SP” or “OB” identifiers was used to indicate the same geographic location. Cases were considered sporadic if health departments did not report the case as outbreak-associated, and the case is without any known epidemiological links to the outbreak cases. Isolate information and sequencing statistics are shown in Supplementary Table 4. NmC isolates from two previously characterized MSM outbreaks, one in Chicago 2003 (n = 5)^6^ and one in Toronto Canada 2001 (n = 6)^51^, were included in the molecular clock analysis along with the isolates of 4 NmC outbreaks from 2009–2015 in this study.

### Molecular typing

Seven MLST loci, *porA* and *fetA* were sequenced using Sanger sequencing or whole genome sequencing. For Sanger sequencing, either the Meningococcal Genome Informatics Platform^52^ or SeqMan Pro (version 12, DNASTAR Lasergene, Madison, WI) was used to analyze the sequencing data. Genomes of meningococcal isolates were sequenced on Illumina sequencer as described below in “whole genome sequencing.” The MLST alleles, *porA*, and *fetA* were identified by BLAST search of alleles in PubMLST against the assembled genomes^53^. Outbreak strain genotype was defined as serogroup:MLST ST/CC:PorA type:FetA type.

### Pulse field gel electrophoresis

Outbreak isolates (n = 81) and a subset of sporadic isolates (n = 51), matched on timeframe and state to outbreak isolates, were prepared for PFGE using *Nhe*I (New England BioLabs, Ipswich, MA) as described by Popovic *et al*.^16^, with one modification: the exclusion of sodium dodecyl sulfate in the plug agarose without causing differences in PFGE patterns^54^. TIFF images were normalized by aligning the gel standard M413 with the database standard using BioNumerics Fingerprinting software version 6.6.11 (Applied Maths, Austin, TX)^16^. After normalization, bands were marked within the range of the standard. Dice coefficients with a 1.5% position tolerance and 1.0% optimization, and unweighted pair group with arithmetic averages (UPGMA) were used to determine the level of similarity between PFGE patterns. Computer analysis was followed by and confirmed with visual inspection. PFGE patterns with the same number of bands were considered indistinguishable if band positions were within 2 kilobases of each other. When a specific PFGE pattern is distinguishable from any existing patterns, a new PFGE pattern type was designated. UPGMA dendrograms based on Dice coefficients were constructed to present PFGE analysis of the outbreak isolates; similarity matrices from these dendrograms were exported for further analysis. Cophenetic correlation was used to determine the reliability of clusters within these dendrograms.

### Whole genome sequencing

Genomic DNA was prepared for sequencing using the 5 Prime ArchivePure DNA Purification kit (Gaithersburg, MD), Ampure (Beckman Coulter Inc.; Indianapolis, IN) as previously described^55^ for sequencing on Pacific Biosystems (PacBio RSII; Menlo Park, CA, USA) and/or Illumina platforms (HiSeq2500 or MiSeq; San Diego, CA, USA); genomic DNA was further prepared for Illumina sequencing with the dual-index NEBNext Ultra DNA library preparation kits (New England Biolabs Inc.; Ipswich, MA)^56^. Genomes generated using PacBio were processed as previously described^56^. The Illumina reads were trimmed with cutadapt^57^ to remove adaptor sequences and the reads below Q28 and 75 bp. *De novo* short read assembly was carried out using SPAdes^58^ with the “-careful” option. The *de novo* assemblies were improved by scaffolding the best contigs using SSPACE^59^ and sequence gaps filled using GapFiller^60^ using short read sequences. This step was implemented using the assembly_improvement pipeline^61^.

### WGS - based analysis methods

Six WGS-based analysis methods were evaluated using the genomic sequences from meningococcal outbreaks and sporadic isolates described in strain collection, above, and are summarized in Table 2.

#### kSNP

The whole genome core SNPs were extracted from the assemblies using kSNP v3.0^62^. The kSNP3 program identifies high-confidence SNPs by matching unique nucleotide sequences (k-mers) from each genome that vary only at the middle site. A k-mer size of 25 bp was implemented and the core SNP alignments from NmB and NmC genomes were used to reconstruct an ML phylogenetic tree using RAxML^63^ with a general time reversible (GTR) nucleotide substitution model, with 100 bootstrap estimates.

#### MASH

MASH (v1.1) was used to calculate distances between the trimmed read sequences of each genome, with parameters k-mer (k) = 32, s = 100000, m = 5, and c = 75. The target coverage (c) and minimum copies (m) of each k-mer were selected to assure that each sketch had an estimated genome size between 2.0 and 2.2 megabases.

#### SNIPPY (High-quality [hq] SNPs)

Trimmed and quality filtered Illumina sequencing reads from NmB and NmC isolates were mapped to serogroup B isolate M18678 and serogroup C isolate M21427, respectively. Both were outbreak isolates included in this study and sequenced using PacBio. Variants in the form of SNPs were called using Snippy v3.1^44,64^, which uses BWA-MEM v0.7.12 for short read mapping. SNPs were called using Freebayes v1.0.2, requiring a minimum read coverage of 10X, and a 90% read concordance at a locus for an SNP to be reported. Snippy generates a core SNP alignment as well as a whole genome SNP alignment. The whole genome alignment was used to infer the ML phylogenies using RAxML as described above. Recombination-adjusted phylogenies for NmC and NmB isolates were generated using ClonalFrameML (CFML)^65^.

#### Parsnp core-genome multi-aligner

Whole genome core alignments were generated for both NmC and NmB isolates using the Parsnp whole genome aligner available through the Harvest suite^66^. The resultant core genome alignment was used to generate ML-based phylogenies using RAxML and for generating the recombination-adjusted phylogenies using ClonalFrameML as described above.

#### Roary core genome comparison

All the genomes were annotated using Prokka v1.8^67^, and the resultant gff3 (general feature format) files were used to perform the pan-genome analysis using Roary^68^ for both NmC and NmB genomes. Roary extracted the coding regions from the input gff3 files and converted them to protein sequences, removed partial sequences and iteratively pre clusters sequences with CD-HIT^69^, which resulted in a reduced set of protein sequences. Then an all-against-all comparison was performed with BLASTP with 95% sequence identity on the reduced protein sequences. Sequences were then clustered with a Markov cluster (MCL) algorithm^70^ based on the BLASTP similarity score. Finally, the pre-clustering results from CD-HIT were merged together with the results of MCL. From the final clustering results, homologous groups containing paralogs were identified based on conserved gene neighborhood information and removed to obtain the true orthologs^68^. In the final step of Roary, core genes were identified and a concatenated core gene alignment was created using PRANK^71^; this alignment was used to generate the ML phylogeny using RAxML and for ClonalFrameML analysis as described above.

#### Core genome MLST (cgMLST)

Genomes of NmB and NmC isolates were uploaded to the Bacterial Isolate Genome Sequence (BIGSdb) genomics platform hosted on the web^53^. These WGS data were compared using the PubMLST Genome Comparator tool that implements a “gene-by-gene” comparative analysis. In brief, a specified set of loci (core genes) among the isolates were compared sequentially to pre-indexed allele identifiers at each core gene and a matrix was generated that contained the allelic distances between NmB and NmC isolates, along with the concatenated core gene alignment^72,73^. The core gene alignment was used to generate the ML phylogeny using RAxML as described above.

In order to quantify similarities between phylogenetic trees reconstructed using various WGS methods, pairwise tree distances were estimated using weighted Robinson – Foulds (wRF) metric^74^. wRF takes into account both the topological similarities as well as branch lengths while calculating the tree distance. All pairwise tree distances were generated using the R package treespace^75^.

### Molecular clock analysis

A timed phylogeny was constructed using BEAST (Bayesian Evolutionary Analysis Sampling Trees)^76^, with genomes of all 24 CC32 NmB and a subset of 71 closely related CC11 NmC isolates that were associated with MSM population. Multiple outbreak strains that belonged to these two CCs caused organizational or community outbreaks over multiple years, providing sufficient temporal signal for molecular clock analysis (root-to-tip correlations estimated using root-to-tip linear regression were 0.3036 for NmB and 0.6842 NmC). The 24 CC32 NmB isolates included all isolates from outbreak OB11 and OB12, 2 OB6 isolates, and 12 sporadic isolates. The 71 CC11 NmC genomes included all isolates from OB7, OB8, and OB14, and 2 OB9 isolates. Six genomes from a well-characterized NmC CC11 outbreak at Toronto in 2001^51^ (obtained from PubMLST^77^) and 5 genomes (sequenced at the CDC) from a MSM outbreak at Chicago in 2003^6^ were also included in the NmC BEAST analysis. For BEAST analysis, first recombination was detected using ClonalFrameML^65^, and the SNP sites determined to be outside of the predicted recombinant segments were used. We used BEAST v1.8.2 with the HKY (Hasegawa, Kishino, and Yan)^78^ substitution model, the coalescent model with constant population size, and a uncorrelated relaxed clock model with an exponential distribution as the prior for the clock rate. Two independent chains of 200 million steps were run for both NmC and NmB alignments, and each was sampled every 1000 steps to ensure good mixing. The results were compared using Tracer to confirm convergence, and the maximum clade credibility tree was generated using Tree Annotator; both tools are provided with the BEAST package^79^.

## Electronic supplementary material

Supplementary Figures

Supplementary Table 1

Supplementary Table 2

Supplementary Table 3

Supplementary Table 4
