# Supplementary material for: Whole genome sequencing for investigations of meningococcal outbreaks in the United States: a retrospective analysis
Source: Sci Rep. 2018 Oct 25;8:15803. doi: 10.1038/s41598-018-33622-5 (PMC6202316; doi:10.1038/s41598-018-33622-5)
Supplement: Supplementary file 1 — Supplementary Figures [file 41598_2018_33622_MOESM1_ESM.pdf]

Supplementary Information

Whole genome sequencing for investigations of meningococcal outbreaks in the United States: a retrospective analysis

Melissa J. Whaley[1]*, Sandeep J. Joseph[1]*, Adam C. Retchless[1], Cecilia B. Kretz[1], Amy Blain[1], Fang Hu[1], How-Yi Chang[1], Sarah A. Meyer[1], Jessica R. MacNeil[1], Timothy D. Read[2], and Xin Wang[1]†

[1]Meningitis and Vaccine Preventable Diseases Branch, Centers for Disease Control and Prevention, Atlanta, Georgia, USA. [2]Division of Infectious Diseases, Department of Medicine, Emory University School of Medicine, Atlanta, Georgia, USA.

Supplementary Figures

Supplementary Figure 1. PFGE dendrogram of NmB outbreak isolates, 2009 - 2015. Labels include isolate ID, clonal complex, PFGE pattern, outbreak ID and year

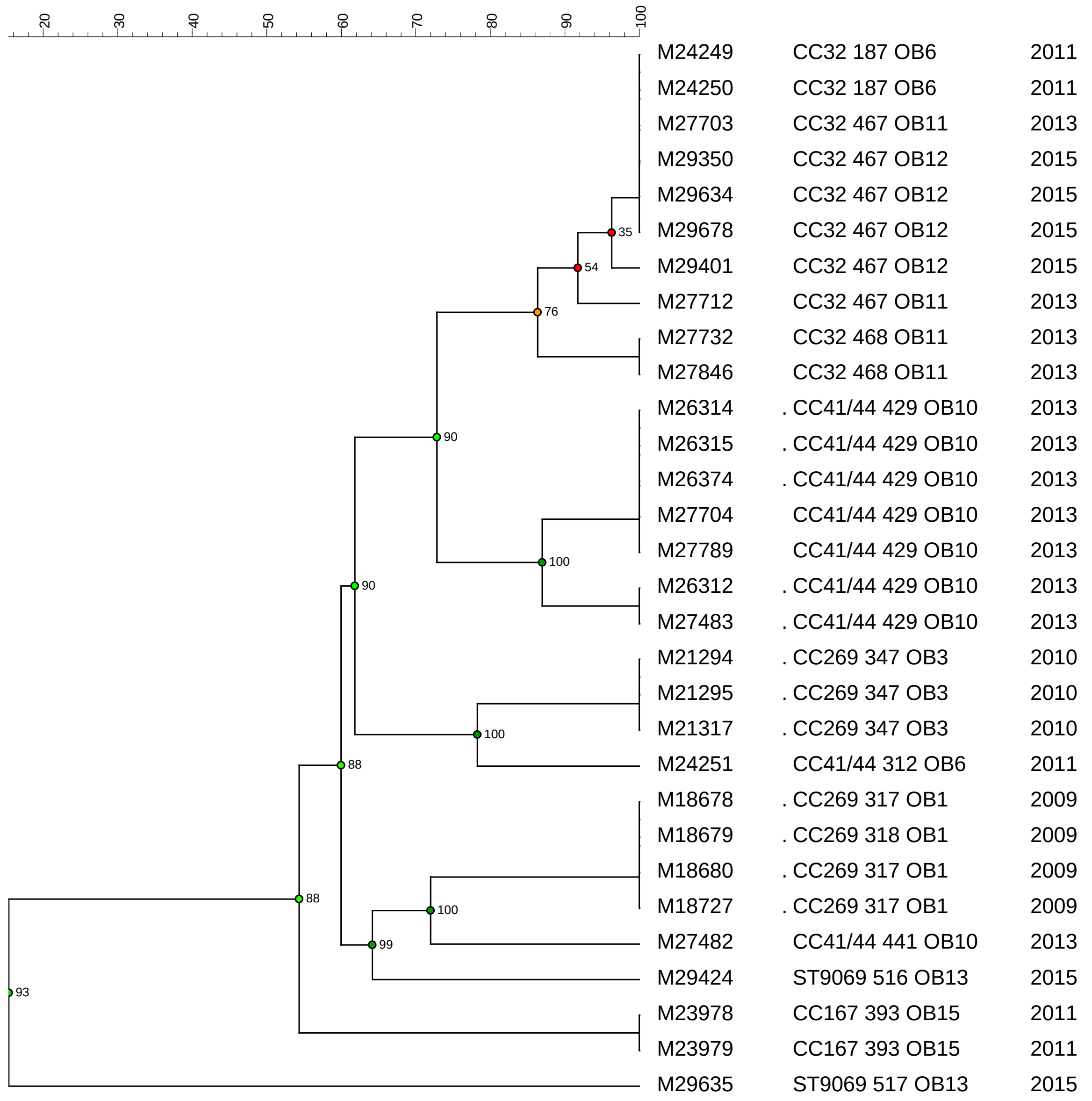

Supplementary Figure 2. PFGE dendrogram of NmC outbreak isolates, 2009 - 2015. Labels include isolate ID, clonal complex, PFGE pattern, outbreak ID and year.

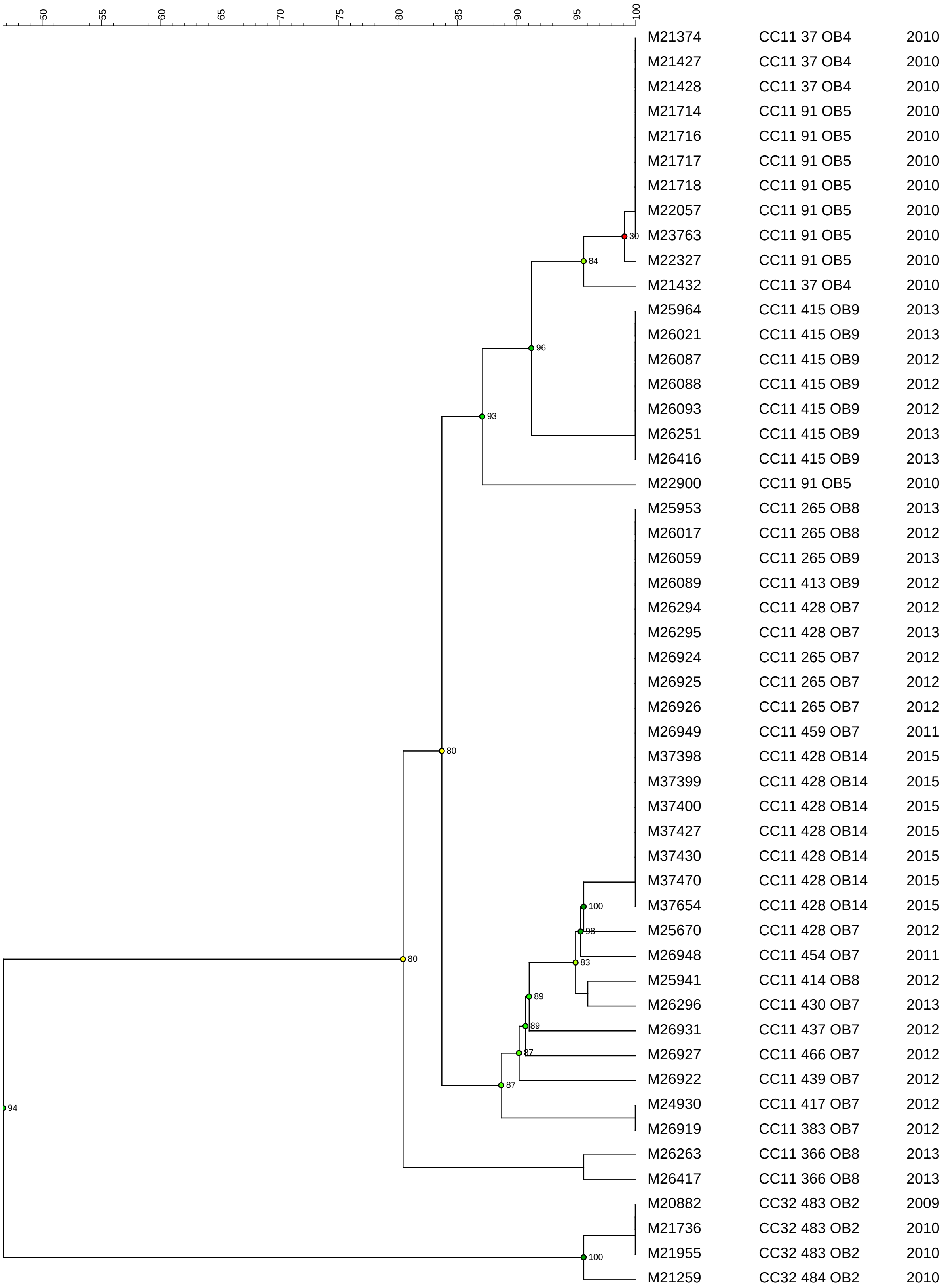

Supplementary Figure 3. **Boxplot showing the distribution of the approximate genetic differences between sporadic and outbreak isolates across different WGS-based analysis methods**. * indicates the group of outbreaks (NmB outbreaks: OB1, OB10, OB13, OB15 and OB3, and NmC outbreaks: OB2, OB4 and OB5) that only clustered together in all the WGS phylogenetic trees and did not cluster with any sporadic isolates. The horizontal line in each boxplot indicates the median SNP/allele differences and the numbers inside the boxplot represents the estimated mean of SNP/allele differences across the different WGS-based analysis methods.

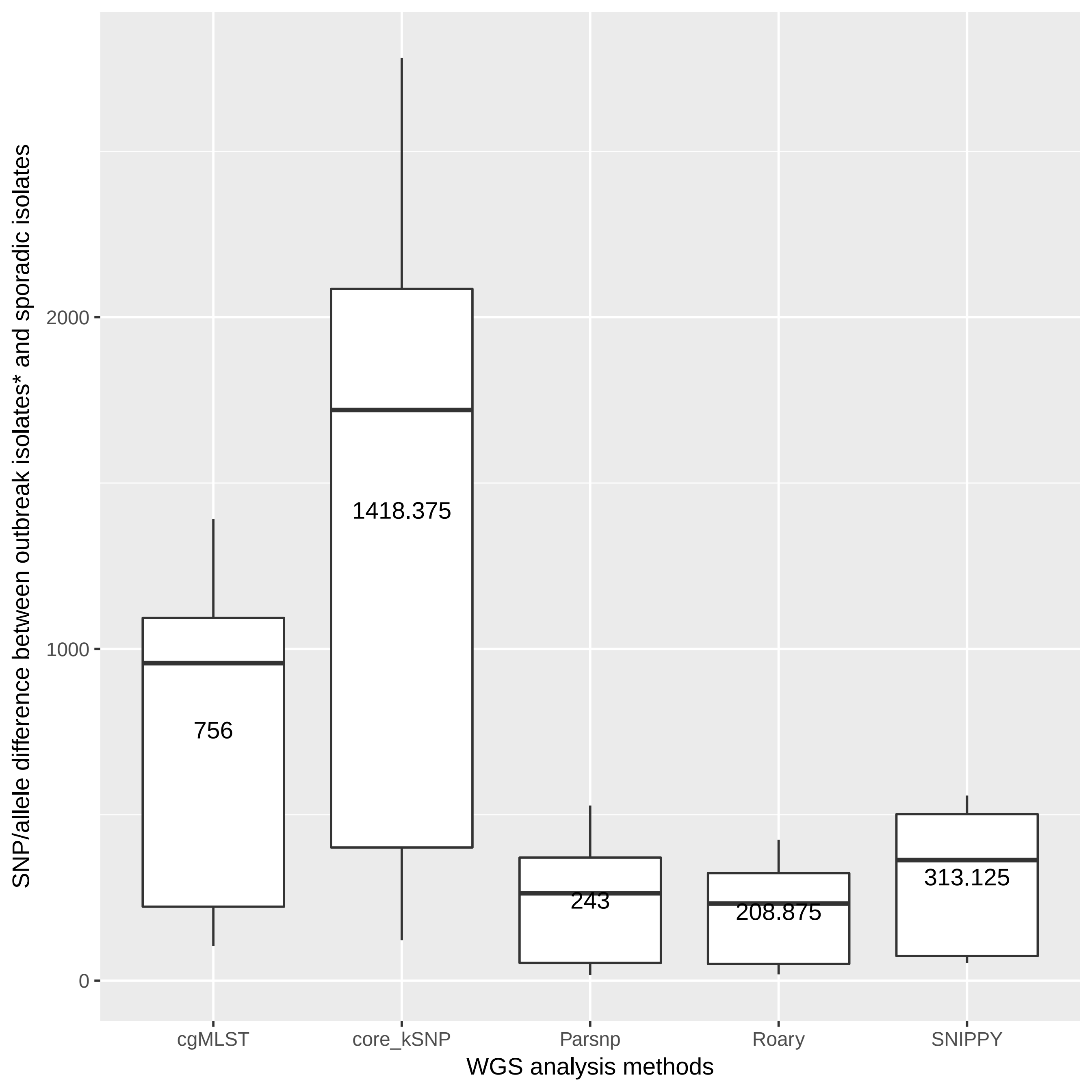
SNP/allele difference between outbreak isolates* and sporadic isolates
2000
1000
0
1418.375
756
243
208.875
313.125
cgMLST
core_kSNP
Parsnp
Roary
SNIPPY
WGS analysis methods
