# Supplementary material for: Whole genome sequencing for investigations of meningococcal outbreaks in the United States: a retrospective analysis
Source: Sci Rep. 2018 Oct 25;8:15803. doi: 10.1038/s41598-018-33622-5 (PMC6202316; doi:10.1038/s41598-018-33622-5)
Supplement: Supplementary file 4 — Supplementary Table 3 [file 41598_2018_33622_MOESM4_ESM.pdf]

Whole genome sequencing for investigations of meningococcal outbreaks in the United States: a retrospective analysis

Melissa J. Whaley[1†], Sandeep J. Joseph[1†], Adam C. Retchless[1], Cecilia B. Kretz[1], Amy Blain[1], Fang Hu[1], How-Yi Chang[1], Sarah A. Meyer[1], Jessica R. MacNeil[1], Timothy D. Read[2], and Xin Wang[1*]

[1]Meningitis and Vaccine Preventable Diseases Branch, Centers for Disease Control and Prevention, Atlanta, Georgia, USA. [2]Division of Infectious Diseases, Department of Medicine, Emory University School of Medicine, Atlanta, Georgia, USA.

Supplementary Table 3. Pairwise weighted Robinson-Foulds tree distance estimates between the phylogenetic trees reconstructed using the WGS analysis methods for NmB and NmC isolates used in this study. Phylogenetic trees inferred from SNIPPY, Parsnp and Roary were corrected for homologous recombination using ClonalFrameML.

**NmB Phylogenetic trees**				
	**SNIPPY**	**Parsnp**	**Roary**	**cgMLST**
**Parsnp**	0.1481			
**Roary**	0.1415	0.00294		
**cgMLST**	5.5085	4.4961	4.4963	
**kSNP**	4.1821	4.1695	4.1695	0.6192
**NmC Phylogenetic trees**				
	**SNIPPY**	**Parsnp**	**Roary**	**cgMLST**
**Parsnp**	0.0026			
**Roary**	0.0020	0.0009		
**cgMLST**	2.0464	2.0450	2.0454	
**kSNP**	2.0933	2.0919	2.0924	0.3624
